# Utilization of a Neonatal Early-Onset Sepsis Calculator to Guide Initial Newborn Management

**DOI:** 10.1097/pq9.0000000000000214

**Published:** 2019-09-23

**Authors:** Bianca M. Leonardi, Margaret Binder, Katherine J. Griswold, Gulgun F. Yalcinkaya, Michele C. Walsh

**Affiliations:** From the * Department of Pediatrics, Akron Children’s Hospital, Akron, Ohio; † Department of Pediatrics, Wake Forest Baptist Health, Winston Salem, N.C.; ‡ Department of Pediatrics, University Hospitals Cleveland Medical Center’Rainbow Babies, Cleveland, Ohio; § Children’s Hospital, Case Western Reserve University School of Medicine, Cleveland, Ohio

## Abstract

Within our hospital system, all infants born to mothers with chorioamnionitis were directly admitted to the neonatal intensive care unit (NICU) for evaluation and treatment of presumed sepsis for a minimum of 48 hours, regardless of clinical appearance. Implementation of a risk-stratification system for thesWe high-risk infants based on the early onset sepsis (EOS) calculator may decrease NICU admissions and antibiotics exposure in well-appearing neonates.

**Methods:**

We used The Model for Improvement as a framework for designing this initiative. Participants were inborn infants 35 weeks and older born to mothers with chorioamnionitis and/or fever. Plan Do Study Act (PDSA) cycles were utilized to educate staff, monitor for sepsis, and follow adherence to the calculator in the newborn nursery.

**Results:**

From June 2015 to June 2016, there were 312 at-risk infants identified and evaluated on the EOS calculator. Of these 312 infants, 228 did not require admission to the NICU based on their risk assessment using the online calculator. Implementation of the Kaiser EOS calculator protocol for at-risk infants decreased NICU admission rates, decreased practitioner practice variability, decreased the number of painful procedures, promoted family bonding, resulted in higher breastfeeding rates at hospital discharge, diminished financial burden, and promoted antibiotic stewardship.

**Conclusion:**

This study demonstrates that the implementation of the sepsis risk calculator at an academic medical center can decrease the number of asymptomatic infants transferred to the NICU for empiric antibiotic treatment.

## INTRODUCTION

“Rule out sepsis” is one of the most common admission diagnoses utilized by neonatal intensive care units (NICUs), despite the low incidence of true culture positive sepsis. Physical examination and laboratory evidence of sepsis in infants are often vague and nonspecific. These include temperature instability, irritability, jaundice, hypoglycemia, poor feeding, bradycardia or tachycardia, respiratory distress, decreased perfusion, lethargy, acidosis, and/or apnea. However, infants who exhibit these signs and symptoms often do not have a true infection.

Early onset sepsis (EOS) occurs within the first 72 hours of an infant’s life. Risk factors for EOS include maternal fever and/or chorioamnionitis, maternal Group B Streptococcus (GBS) colonization, inadequate intrapartum antibiotic prophylaxis for GBS, preterm labor/premature rupture of membranes, and prolonged rupture of membranes. EOS has a reported incidence of 0.5–1.2 per 1,000 live births. However, many large academic institutions report evaluation and treatment of up to 5–15% of all live births.^1,2^

In the 1990s, CDC consensus guidelines recommended universal screening for maternal GBS colonization and maternal intrapartum antibiotic prophylaxis. As a result of widespread implementation of these guidelines, the burden of GBS-related EOS decreased by over 80% (from 1 to 2 per 1,000 live births to 0.34–0.37 per 1,000 live births).^2–4^ Universal screening, unfortunately, led to an increase in over 30% in the number of infants exposed to intrapartum antibiotics. This increased percentage raises concern for the development of antimicrobial resistant pathogens and increased incidence of EOS resulting from Gram-negative species.^2,3^

Chorioamnionitis is reported to complicate 35–4% of all deliveries and is a known risk factor for the development of EOS.^3^ The presence of chorioamnionitis is primarily a clinical diagnosis based upon Gibbs’ criteria, which requires the presence of maternal fever >38^o^C and two or more of the following: maternal tachycardia (*>*100 beats/min), fetal tachycardia (*>*160 beats/min), maternal leukocytosis (*>*15,000 cells/mm^3^), purulent or foul-smelling amniotic fluid, and/or uterine/fundal tenderness.^5,6^ Current guidelines from the CDC and the Committee on Fetus and Newborn for the prevention of perinatal GBS disease recommend laboratory evaluation (CBC and blood culture) and empiric antibiotic treatment for 48 hours of infants born to mothers with chorioamnionitis.^5,7,8^

To reduce the administration of antibiotics to well-appearing infants in the immediate postnatal period, researchers at Kaiser developed a multivariate predictive model to estimate the risk of EOS in infants 34 weeks and older’ gestation.^1,9^ This model utilizes maternal antepartum risk factors coupled with an infant’s clinical appearance at birth and over the first 12 hours of life to predict the risk for EOS. The online calculator allows the user to select a baseline rate of early onset neonatal sepsis; for this study, we used the CDC rate of 0.5 per 1,000 live births. Kaiser has published an online EOS calculator for use by other institutions.^1,9^

Historically, at MacDonald Women’s and Rainbow Babies and Children’s Hospital, infants 35 weeks and older’ gestation born to mothers diagnosed clinically with chorioamnionitis were admitted to the NICU regardless of clinical appearance. In 2013 and 2014, approximately 5% of women delivering at 35 weeks and older’ gestation had a diagnosis of chorioamnionitis. All these infants underwent a standard neonatal septic evaluation requiring laboratory work and peripheral IV placement and were treated empirically with ampicillin and gentamicin for a minimum of 48 hours.

**Fig. 1.**
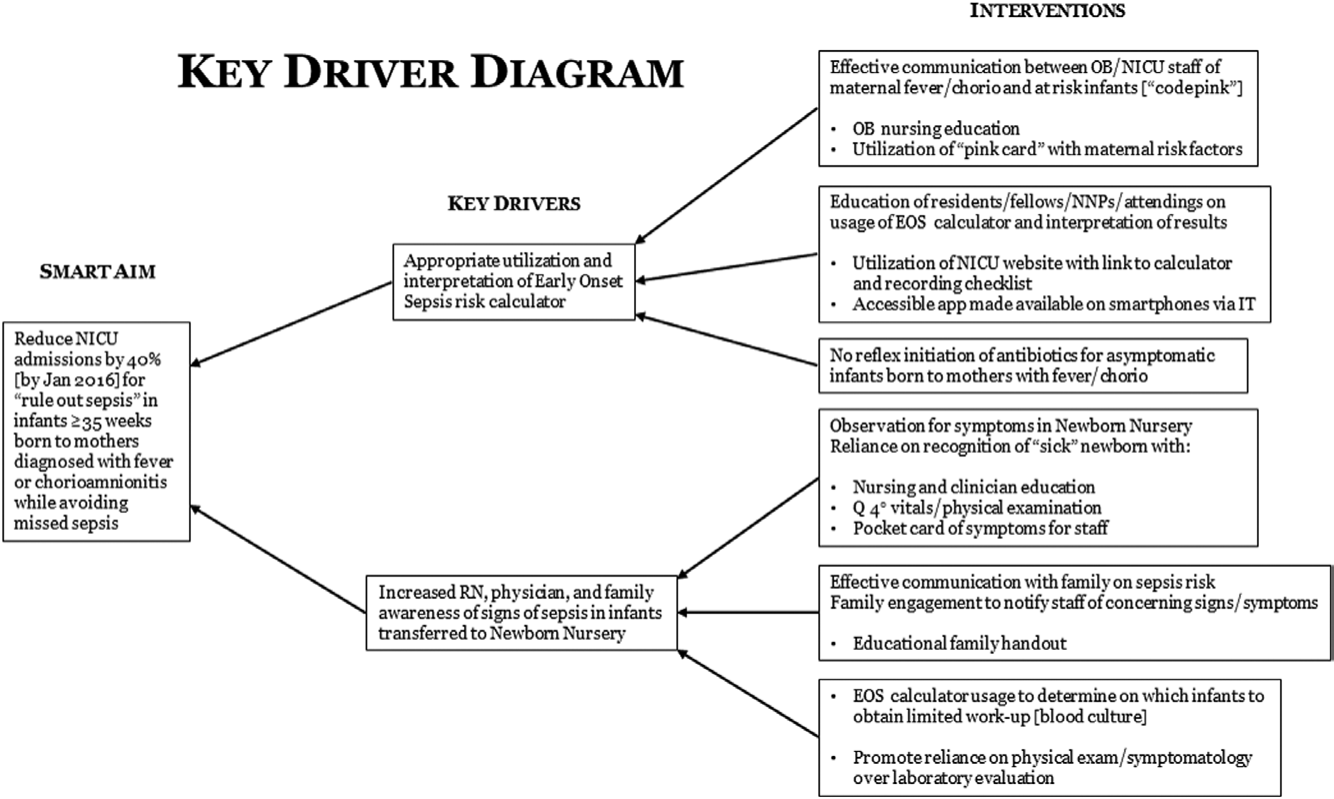
Key driver diagram. The Model for Improvement was utilized as a framework for designing this multi-disciplinary initiative with several PDSA cycles. OB, obstetrics; RN, registered nurse.

The goal of this quality improvement initiative was to reduce NICU admissions by 40% for well-appearing infants older than 35 weeks born to mothers with fever and/or chorioamnionitis. We utilized the Kaiser EOS calculator to risk-stratify newborns based on maternal antepartum risk factors and neonatal clinical exam. Figure 1 shows the key driver diagram for this project.

## METHODS

This study was approved by the IRB as a quality improvement project at University Hospitals Cleveland Medical Center in Cleveland, Ohio.

The quality improvement team was composed of physicians in charge of the newborn nursery, NICU attendings, a pharmacist, NICU nursing leadership, and a medical student.

We placed infants born 35 weeks and older’ gestation to mothers with fever and/or clinically diagnosed chorioamnionitis on the Kaiser EOS calculator. The calculator is accessible on the following website: https://neonatalsepsiscalculator.kaiserpermanente.org/. We used the CDC national incidence of EOS (0.5 per 1,000 live births).

This quality improvement project implemented several PDSA ramps; each ramp involved several PDSA cycles. The first PDSA ramp required proper notification of the pediatric team by Labor and Delivery (L&D) staff of infants at risk for EOS. To facilitate communication of EOS risk factors, we developed a “pink card,” which outlined maternal antepartum risk factors for EOS. This card was given to the pediatric team promptly following delivery. We tracked the proper notification of the pediatric team and frequency/accuracy of pink card utilization by the L&D staff. The primary investigator manually tracked pink card utilization.

The second PDSA ramp focused on proper utilization of the EOS calculator. Pediatric residents, neonatal fellows, and attendings received education on the use of the EOS calculator. These caregivers were responsible for using the online calculator following the delivery of an identified at-risk infant and examining the neonate to determine the need for laboratory work and/or antibiotics. The guidelines were posted in the nursery and made into laminated cards for clinicians’ badges. Infants deemed ill at birth were immediately transferred to the NICU for laboratory evaluation and antibiotic administration.

Infants who were considered well and remained in the newborn nursery were followed with vital signs every 4 hours to evaluate for physiologic abnormalities. Parents received a hospital approved patient information handout on signs of neonatal infection. We instructed parents to alert caregivers with concerns about their newborn. Nursing staff notified the responsible caregiver of any infant on the EOS calculator with a vital sign abnormality or other concerns. This notification prompted the clinician to evaluate the infant and determine if further laboratory evaluation, transfer to NICU for antibiotic therapy, or continued observation were warranted. We tracked proper utilization of the EOS calculator by the completion of an “EOS checklist” by these clinicians. For infants transferred to the NICU, either at birth or following an initial newborn nursery stay, we tracked parameters related to the rationale for transfer, administration of antibiotics, laboratory evaluation and results, duration of admission, and intended feeding method at birth and discharge.

The next PDSA ramp focused on additional methods to avoid missing sepsis. Our hospital is designated “Baby Friendly,” so every baby evaluated with the EOS calculator roomed-in with their mother for the duration of the newborn hospitalization. During this period, nursing staff monitored the newborn’s vitals every 4 hours for a minimum of 48 hours. The parents of each baby placed on the EOS calculator were required to have identified and scheduled a follow-up appointment with a pediatric provider within 24–48 hours of discharge.

We conducted weekly audits for infants tracked on the EOS calculator as an additional safety monitoring tool. Monthly reports tracking these infants’ nursery admissions, along with transfers, antibiotic utilization rates, and cases of culture positive or clinical sepsis, were generated and reviewed by the physicians performing this quality improvement project.

### Context

Rainbow Babies and Children’s Hospital located in Cleveland, Ohio, has an 82-bed level IV academic NICU with approximately 1,200 annual admissions. The adjacent delivery hospital, MacDonald Women’s Hospital, has 4,000 deliveries per year of infants older than 35 weeks gestation. During the study period, there was minimal fluctuation in the number of admissions to the newborn nursery. Of these deliveries, 200 are affected by chorioamnionitis, and 75 are affected by maternal fever without chorioamnionitis.

### Measures

The primary outcome measure was the monthly rate of NICU admissions for sepsis evaluation/treatment in infants older than 35 weeks gestation born to mothers with chorioamnionitis. Secondary outcomes included monthly rates of sepsis amongst at-risk infants and breastfeeding rates at discharge for infants admitted to the NICU as compared with those who remained in the newborn nursery.

Process measures include the monthly rate of neonatal team notification of at-risk infants by the L&D staff, the monthly rate of pink card utilization by the L&D staff, the monthly rate of proper EOS calculator usage, and the clinical indications for transfer at birth and subsequently to NICU infants tracked by the EOS calculator.

Balancing measures included the number of infants per month who were well at birth and became equivocal or clinically ill with sepsis requiring transfer to the NICU and the monthly rate of readmission for sepsis.

## RESULTS

**Fig. 2.**
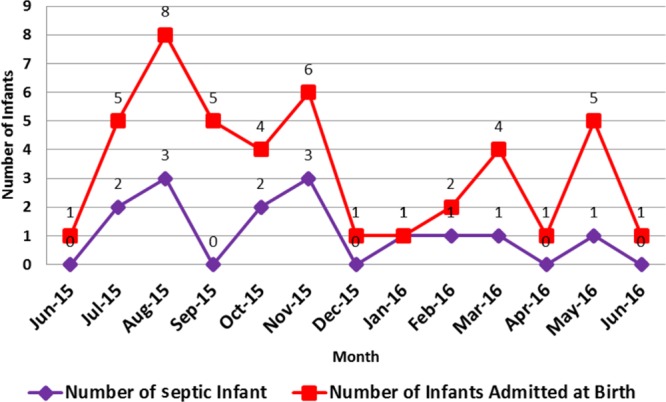
Incidence of sepsis in symptomatic infants admitted to the NICU for evaluation and empiric treatment incidence of sepsis among infants symptomatic at birth. Sepsis is defined as a positive blood or CSF culture or clinical illness necessitating >48 hours of antibiotic therapy. There was a single case of bacteremia during this time.

From June 2015 to June 2016, there were 312 at-risk infants identified and evaluated by the EOS calculator. Of these 312 infants, 44 (14%) were symptomatic at birth prompting transfer from the delivery room to the NICU. An additional 40 infants (12.8%) became symptomatic and required later transfer to the NICU. The remaining 228 infants did not require admission to the NICU based on their risk assessment using the online calculator. Of those requiring NICU admission directly from the delivery room, 68% presented with respiratory distress at birth or shortly after that. Figure 2 shows the number of infants with culture-proven or presumed sepsis. Most symptomatic infants admitted for sepsis evaluation did not have culture-proven sepsis or require prolonged antibiotics.

**Fig. 3.**
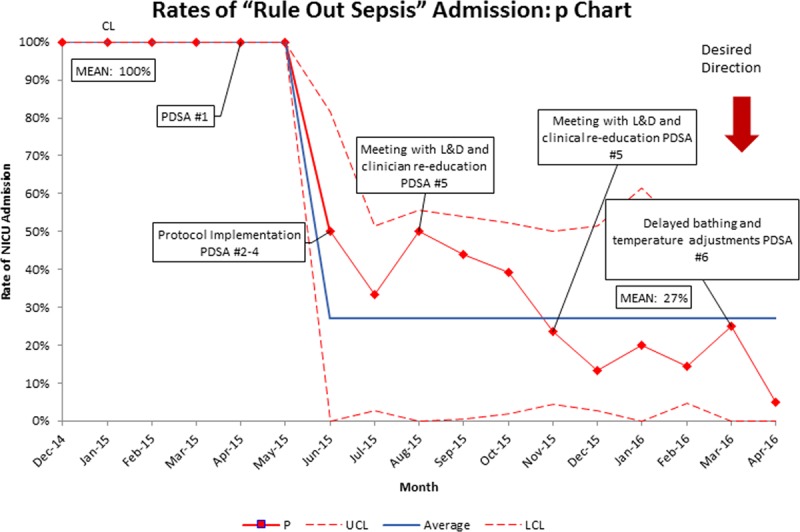
Incidence of sepsis in well infants who became symptomatic incidence of sepsis amongst infants initially well who developed equivocal vitals or clinical illness. Sepsis is defined as a positive blood or CSF culture or clinical illness necessitating >48 hours of antibiotics therapy. There were no positive blood cultures during this time. LCL, lower control limit; UCL, upper control limit.

**Fig. 4.**
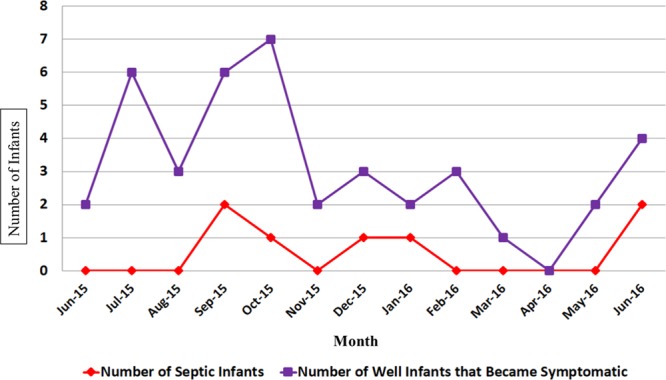
“Rule-out-sepsis” admission/treatment rates and rates of sepsis. Overall admission rates for “rule-out sepsis” evaluation and treatment in infants older than 35 weeks born to mothers who were diagnosed with chorioamnionitis or fever and the overall rates of sepsis. Sepsis is defined as a positive blood or CSF culture or clinical illness necessitation >48 hours of antibiotics therapy. There was a single case of culture-positive sepsis during this time.

Over half of the initially well-appearing infants (54%), who subsequently required transfer to the NICU, presented with temperature instability. This finding prompted the study team to evaluate immediate newborn care upon admission to the nursery. At the start of the study, the initial newborn bath occurred upon admission to the postpartum unit. Even infants who are not at risk for sepsis may have temperature instability after their first bath. Therefore, the decision was made to delay initial bathing until 24 hours of life (Fig. 3; PDSA cycle 6). This practice change dramatically decreased the number of infants requiring late transfer to the NICU. Figure 4 shows the incidence of sepsis in initially well-appearing infants who subsequently required transfer to the NICU. Of the initially asymptomatic infants who later transferred to the NICU for sepsis evaluation, there were no positive blood cultures. Only 7 infants with presumed sepsis got treatment with prolonged courses of antibiotics.

Figure 3 shows the overall admission rates for “rule out sepsis” evaluation and treatment in infants older than 35 weeks gestation born to mothers with chorioamnionitis or fever. Sepsis is defined as a positive blood or CSF culture or clinical illness requiring >48 hours of antibiotic therapy.

Breastfeeding rates at discharge were 89% for infants remaining with their mothers in the newborn nursery. In contrast, only 37% of infants requiring NICU admission for sepsis evaluation and treatment were breastfeeding at discharge.

We followed the study population for readmission within our hospital system following hospital discharge to ensure that there were no cases of “missed sepsis.” Over the 12 months of the study period, 5 infants got readmitted within 30 days of hospital discharge. Of these infants, none was diagnosed with bacterial sepsis. We looked at all hospital readmissions regardless of readmission diagnosis to exclude cases of missed sepsis.

## DISCUSSION

In this study, implementation of the EOS calculator decreased NICU admissions for “rule out sepsis” in well-appearing infants born to mothers with fever or chorioamnionitis. It also reduced antibiotic utilization rates in this population, which before the implementation of the EOS calculator was 100% for newborns born to mothers with a diagnosis of chorioamnionitis. Close monitoring of newborn vital signs and clinical examination enabled well-appearing infants to remain in the newborn nursery. A postdischarge appointment with a pediatric care provider within 1–2 days of hospital discharge was enforced to ensure adequate follow-up. Nurse Practitioners called families if they missed their appointments to ensure adequate follow-up.

Implementation of the Kaiser EOS calculator protocol for at-risk infants decreased NICU admission rates, decreased practitioner practice variability, decreased the number of painful procedures, promoted family bonding, diminished financial burden to the patient/hospital/community, and promoted antibiotic stewardship. We attribute significantly higher breastfeeding rates at newborn discharge to rooming-in with the mother for the duration of the newborn hospitalization. Therefore, we prevented disruption of the mother–infant dyad by implementing the sepsis calculator. Before using the Kaiser EOS calculator, some providers would treat based on screening laboratory results (such as CBC and CRP), whereas other providers would empirically treat all newborns with exposure to maternal chorioamnionitis.

One of the primary practice changes that were implemented as a result of this quality improvement initiative was the delay in newborn bathing until 24 hours of life in the newborn nursery. Before this study, nursing staff bathed newborns around 4 hours of life upon admission to the postpartum floor. Many babies (even those without risk factors for infection) would have temperature instability following the bath; thus triggering a sepsis evaluation. Following the introduction of delayed bathing, there was a dramatic decrease in the number of infants at risk for sepsis requiring transfer to the NICU for temperature instability.

Laboratory evaluation of well-appearing infants can result in nonspecific findings. This testing may lead a clinician to empirically treat laboratory values rather than base treatment on the clinical appearance of the infant. The evaluation of clinically well-appearing infants requires painful procedures such as blood draws, peripheral IV access, lumbar punctures, and exposure to broad-spectrum antibiotics early in life.^10^ The evaluation and treatment of well-appearing infants born to mothers with chorioamnionitis may lead to the unnecessary disruption of the parental–infant dyad, decreased early family bonding, and difficulty with breastfeeding initiation. There is also a significant economic burden to the healthcare system of treating well-appearing infants with antibiotics in a NICU environment.

Antibiotic administration in early infancy is not without risk. There is an association between exposure to broad-spectrum antibiotics during the first week of life and wheezing in infancy and early childhood.^6,11^ Antimicrobial resistance stemming from unnecessary usage is of growing concern.^12^ Studies link increased body mass index and the incidence of obesity in children to the early use of broad-spectrum antibiotics within the first 6 months of life.^13,14^ The intestinal microbiome is disrupted following antibiotic administration to mice pups, with resultant development of obesity and diabetes, providing a proposed mechanism in children.^12^ Alterations in the intestinal microbiome can lead to the development of allergy and atopy.^15^

### Limitations

We conducted this study at a single academic medical center with a high-risk delivery service and attached Level IV NICU, which may limit its broader application. In this setting, we can continuously monitor and/or perform frequent nursing assessments. This high level of patient observation may not be available in all nurseries. However, any nursery can apply the lessons learned when caring for newborns at risk for EOS.

We only tracked newborn readmissions within our hospital system. Therefore, we do not have data related to out-of-system hospital readmissions.

## CONCLUSIONS

This study demonstrates that the implementation of the sepsis risk calculator at an academic medical center can decrease the number of asymptomatic infants transferred to the NICU for empiric antibiotic treatment. Review of all infants placed on the calculator showed no missed sepsis and no readmissions for EOS.

## DISCLOSURE

The authors have no financial interest to declare in relation to the content of this article.
